# Thermosensitive hydrogel loaded with concentrated growth factors promote bone repair in segmental bone defects

**DOI:** 10.3389/fbioe.2022.1039117

**Published:** 2022-11-01

**Authors:** Yuxin Zhang, Tianchang Wang, Dahe Zhang, Jiayi Li, Xiaokun Yue, Weiqing Kong, Xiaoding Gu, Zixian Jiao, Chi Yang

**Affiliations:** ^1^ Shanghai Key Laboratory of Stomatology, National Center for Stomatology, Department of Oral Surgery, Shanghai Ninth People’s Hospital, Shanghai Jiao Tong University School of Medicine, College of Stomatology, National Clinical Research Center for Oral Diseases, Shanghai Jiao Tong University, Shanghai, China; ^2^ Department of Rehabilitation Medicine, Huangpu Branch, Shanghai Ninth People’s Hospital, Shanghai Jiao Tong University School of Medicine, Shanghai, China; ^3^ Shanghai Key Laboratory of Orthopaedic Implant, Department of Orthopaedic Surgery, Shanghai Ninth People’s Hospital, Shanghai Jiao Tong University School of Medicine, Shanghai, China; ^4^ Department of Spinal Surgery, The Affiliated Hospital of Qingdao University, Qingdao, China

**Keywords:** bone marrow mesenchymal stem cells, concentrate growth factor, hydrogel, bone defects, bone generation

## Abstract

Treating critical-size bone defects beyond the body’s self-healing capacity is a challenging clinical task. In this study, we investigate the effect of concentrate growth factors (CGFs) loaded Poloxamer 407 hydrogel on the viability and osteogenic differentiation potential of bone marrow mesenchymal stem cells (BMSCs) and reconstruction of critical-size bone defects. *In vitro*, this CGFs-loaded thermosensitive hydrogel can significantly promote proliferation, maintain cell viability, and induce osteogenic differentiation of BMSCs by up-regulating the mineralization and alkaline phosphatase (ALP) activity, as well as gene markers, including runt-related transcription factor-2 (*Runx-2*), type I collagen (*Col-1*), osteocalcin (*OCN*), as well as osteopontin (*OPN*). *In vivo*, Micro-CT radiography analysis and histological detection demonstrated that the CGFs-loaded hydrogel significantly induced bone healing and reconstructed the medullary cavity structure in critical-size bone defect models. In conclusion, this strategy of transplantation of CGFs-loaded hydrogel promoted bone regeneration and prevented bone nonunion, so as to provide basis for clinical treatment for repairing critical-size bone defects.

## Introduction

Repairing of critical-size bone defects is an arduous challenge for orthopedic surgeons. Because the defect exceeds the body’s ability of self-healing, coupled with the lack of advanced treatment to promote bone regeneration, the insufficient bone repair will result in some serious complications, including nonunion, bone atrophy, and bone deformities (Angerame et al., 2019; Wang et al., 2019; Li et al., 2022a; Yang et al., 2022). Clinically, autologous bone graft is considered to be the gold standard, but limited by inadequate donor supply, complicated surgical procedures, as well as potential donor pain and infection (Wu et al., 2019). Furthermore, allogeneic bone graft is hindered by ethical issues, high costs. Conventional bone graft substitutes often exhibit weak osteoinductive abilities and tend to triggering inflammatory reactions, resulting in inadequate bone repair in large bone defects (Baldwin et al., 2019). Therefore, the construction of a novel and efficient treatment strategy, which can enhance critical-size bone defects repair, would be of great significance to deal with these challenges.

Attributed to the high self-replication ability, easily isolated, low immunogenicity, and osteogenic differentiation potential, bone marrow mesenchymal stem cells (BMSCs) are regarded as critical endogenous materials to promote bone repair (Guo et al., 2020). However, how to induce rapid proliferation and sufficient osteogenic differentiation of limited BMSCs around the bone defects is still a challenge in clinical practice, which is vital for bone repair in critical-size bone defects (Li et al., 2022b; Li et al., 2022d). Considering the important role of various growth factors in osteogenic differentiation of BMSCs, the introduction of multiple classes and cascaded release of bioactive molecules may be a potential strategy to accelerate bone regeneration. Platelets are a natural source of multiple growth factors, including platelet-derived growth factor (PDGF), vascular endothelial growth factor (VEGF), fibroblast growth factor (FGF), and insulin-like growth factor (IGF), transforming growth factor β1 (TGF-β1), TGF-β2, and so on, which play an important role in promoting cell proliferation, differentiation, tissue regeneration and repair (Intini, 2009). Concentrated growth factor (CGFs) is a third-generation platelet concentrate extracted from blood, which was developed by Sacco in the year 2006 (Lokwani et al., 2020; Li et al., 2021a). Previous study reported that CGFs contains more abundant growth factors than the other platelet-based products, e.g., platelet-rich plasma (PRP) or platelet-rich fibrin (PRF) (Qiao et al., 2017). The previous study has demonstrated that the potential beneficial effects of CGFs in inducing osteogenic differentiation and enhancing bone repair by up-regulating the ALP activity and OCN levels (Sahin et al., 2018; Wang et al., 2020).

In this study, a thermosensitive hydrogel crosslinked by Poloxamer 407, was used to incorporate CGFs to prepare a local sustained-release system with injectable property to enhance critical-size bone defects healing and prevent the occurrence of nonunion. In general, this CGFs-loaded hydrogel containing multiple growth factors could significantly promote BMSCs proliferation and osteogenic differentiation, and induce bone healing in the critical-size bone defects of radius in rabbit models, thus providing an alternative strategy to prevent bone nonunion in clinical.

## Materials and methods

### Materials

Poloxamer 407 was supplied by Bayee Chemical Co., Ltd. (Hangzhou, China). Low glucose Dulbecco’s Modified Eagle Medium (DMEM), fetal bovine serum (FBS), streptomycin–penicillin, and 0.25% trypsin EDTA were obtained from Gibco® Life Technologies (CA, United States). For cell experiments, the rabbit BMSCs were purchased from ATCC Co., Ltd. (Maryland, United States). Enzyme linked immunosorbent assay (ELISA) Kits of TGF-β was supplied by ZCIBIO Technology Co.,Ltd. (Shanghai, China) and VEGF was obtained from Hengyuan Biotechnology Co., Ltd. (Shanghai, China). Calcein-AM/Propidium Iodide (PI), Cell Counting Kit-8 (CCK-8) assay, BCA Protein Assay Kit, Alkaline Phosphatase (ALP) Kit, and RIPA Lysis Buffer were purchased from Beyotime Biotechnology (Shanghai, China). Osteogenic induction medium of rabbit BMSCs and stain of Alizarin Red were supplied by Cyagen Biosciences (CA, United States). Eastep Super Total RNA Extraction Kit was supplied by Promega (Shanghai, China). Perfect Real Time RT reagent kit was obtained from Takara Bio (Dalian, China). For histological staining, hematoxylin eosin (H&E) stain was purchased from Thermo Fisher Scientific (MA, United States). For immunofluorescence staining, primary antibodies were supplied by Abcam (Cambridge, United Kingdom), and secondaries antibodies were supplied by Jackson ImmunoResearch Laboratories (West Grove, PA, United States).

### CGFs preparation

For the experiments, 5 ml of venous blood was drawn from the lateral vein of the right upper limb with a 24-gauge needle of the New Zealand white rabbits under general anesthesia. The process CGFs preparation was according to previous reports (Sahin et al., 2018; Wang et al., 2020). In brief, the blood was directly injected into a sterile vacuette tubes without anticoagulants (Greiner Bio-One, GmbH, Kremsmunster, Austria), and then centrifuged (Thermo Scientific SL1R Plus, Thermo Fisher Scientific, MA, United States) for 13 min to prepare CGFs. After centrifugation, the blood samples were divided into three layers, namely the upper platelet poor plasma (PPP) layer, the middle yellow layer consisting of CGFs and aggregated platelets, and the lower red blood cells layer. The middle layer was used for further analysis.

### Hydrogels preparation and characterization

To prepare the thermosensitive hydrogel, Poloxamer 407 powder was dissolved by 0.01 M PBS (pH = 7.4) with the ratio of 25%:75% (w/w). Subsequently, the Poloxamer 407 solution was left overnight at 4°C to fully dissolve the powder and attain a transparent solution. And then, increasing the temperature to 37°C, the solution transformed to gel state. The thermosensitive property of the hydrogel was conducted by a rheometer (Malvern, United Kingdom) from 0°C to 40°C. Moreover, the morphology was detected through a JSM-6700F scanning electron microscope (SEM) (JEOL, Japan) after freeze-drying.

To prepare the CGFs-loaded hydrogel, CGFs was added into the Poloxamer 407 solution at a ratio of 1:3 at 4°C. Fully mix and raise the temperature to 37°C, CGFs-loaded hydrogel was prepared. To detect the sustained-release behavior of hydrogel, 1 ml CGFs-loaded hydrogel (containing 0.75 ml Poloxamer 407 and 0.25 ml CGFs) and 0.25 ml CGFs were put in sterile bottles and immersed in 5 ml PBS at 37°C, respectively. At scheduled time intervals, 5 ml medium was collected and replaced with another 5 ml fresh PBS. The collected PBS was stored at −80°C for subsequent factors release analysis. Concentration of growth factors, including TGF-β and VEGF, in release solutions were measured by ELISA Kits according to the manufacturer’s instructions. The absorbance of samples was measured by a Multiskan EX microplate reader (Thermo Fisher Scientific, MA, United States) at 450 nm within 15 min after adding the termination solution. The cumulative release curves of growth factors were calculated according to the standard curve.

### Cell viability assays

CCK-8 detection and Calcein-AM/PI staining were carried out to evaluate the biocompatibility of prepared hydrogel and the effect of cell viability of CGFs-loaded hydrogel. Briefly, BMSCs were seeded in 24-well plates at the density of 2 × 10^4^/well. The plates coated with hydrogel and CGFs-loaded hydrogel in advance, abbreviated as Gel group and CGF/Gel group, and BMSCs seeded in the blank plates were regarded as control group (abbreviated as Con). The cells with different treatments were cultured with low glucose DMEM containing 10% FBS (v/v), 1% penicillin and streptomycin in an incubator (Thermo Fisher Scientific, MA, United States) with 37°C and 5% CO_2_ atmosphere. Cell proliferation assays were detected by a CCK-8 kit on the 1, 4, and 7 days. In brief, 10% CCK-8 working solution was added to the cell samples receiving different treatments and reaction for 2 h in an incubator. Subsequently, 100 μl solution from different samples was transferred into the 96-well plates as the detection solution and analyzed at 450 nm by microplate reader. The absorbances of each sample were evaluated at 450 nm by a microplate reader (Multiskan EX, Thermo Fisher Scientific, MA, United States). To study cell survival, Calcein-AM/PI staining of several cell samples was carried out after 3 days of treatment. Say concretely, 2 μM Calcein-AM mixed with 4.5 μM PI were transferred into each well, and then the cell samples were incubated for 15 min at 37°C in the dark. The fluorescent images were observed by a FV1000 confocal laser scanning microscope (CLSM) (Olympus, Japan). The percentage of survival rate of BMSCs was calculated by ImageJ software.

### *In vitro* osteogenic induction of hydrogels

BMSCs were seeded into the 24-well plates with 2 × 10^4^ cells/well as in above cell experiments. After 24 h of incubation, the cells adhered to the wall, and then the DMEM was replaced by osteogenic induction medium for incubating with 7 and 14 days, respectively. The ALP activity was detected according to the manufacturer’s protocols of the ALP Assay Kit. Briefly, after washed by PBS for 3 times, the cells were lysed by RIPA Lysis Buffer on ice. The concentration of total protein was evaluated by the BCA Protein Assay Kit. The optical density (OD) of ALP protein was detected at 520 nm. Finally, the ALP activity was represented by ALP level normalized to the total protein. In addition, to evaluate the formation of calcium nodules, alizarin red staining was performed. Briefly, after fixation with 4% paraformaldehyde for 10 min, the BMSCs were washed with PBS and then stained with 0.1% alizarin red for 30 min at room temperature. After appearance observation of mineralization, 10% cetylpyridinium chloride (1 ml) was added into the samples to dissolve the stained mineralized nodules for 30 min at room temperature for subsequent semi-quantitative analysis by the Microplate Reader at 562 nm.

### Real-time quantitative PCR

The expression level of osteogenic differentiation genes, such as runt-related transcription factor-2 (*Runx-2*), type I collagen (*Col-1*), osteocalcin (*OCN*), and osteopontin (*OPN*) were detected by RT-qPCR. The amplification and evaluation of RT-qPCR was conducted by a LightCycler 480 through 2× Fast SYBR Green Master Mix (Roche Diagnostics, Basel, Switzerland). The levels of these mRNAs expression were normalized by GAPDH and calculated according to the 2^−ΔΔCt^ method. The primers sequences were listed in Table 1.

**TABLE 1:** Primer sequences of genes.

Gene	Oligonucleotide primers (5′-3′)
*Runx-2*	F: 5′-ACT​ACC​AGC​CAC​CGA​GAC​CA-3′
	R: 5′-ACT​GCT​TGC​AGC​CTT​AAA​TGA​CTC​T-3′
*Col-1*	F: 5′-TCC​GGC​TCC​TGC​TCC​TCT​TA-3′
	R: 5′-GGC​CAG​TGT​CTC​CCT​TG-3′
*OCN*	F: 5′-AGC​CAC​CGA​GAC​ACC​ATG​AGA-3′
	R: 5′-CAGGGGATCCGGGTAAGGA-3′
*OPN*	F: 5′-GCT​AAA​CCC​TGA​CCC​ATC​T-3′
	F: 5′-CGT​CGG​ATT​CAT​TGG​AGT-3′
*GAPDH*	F: 5′-CAA​TGA​CCC​CTT​CAT​TGA​CC-3′
	R: 5′-TGG​ACT​CCA​CGA​CGT​ACT​CA-3′

### Animals’ surgical procedures

The New Zealand white rabbits (female, five-month-old) were applied to prepare critical-size bone defect models under general anesthesia by 3% (w/v) pentobarbital at the dose of 50 mg/kg. Briefly, the right lateral radial incision was chosen to expose the radius by separating the tissue layer by layer after skin sterilizing. Subsequently, 15 mm segmental bone defects were made on the right lateral radial by a bone saw, and the bone debris in the defects and periosteum around the end of bone incision was removed. After washing, these rabbits receiving different treatments were divided into three groups, namely, Con group without treatment, and Gel group and CGF/Gel group filled with 1 ml hydrogel and CGFs-loaded hydrogel, respectively. The tissues were sutured layer by layer to close the incision.

At 6 weeks and 12 weeks after implantation, the experimental animals were sacrificed by pentobarbital sodium at a dose of 100 mg/kg intravenously. All rabbits were intramuscular administration of calcein (8 mg/kg) on 14 and 4 days before sacrifice. The right radius samples were collected and stored at −80°C for sequential RT-qPCR detections. The remaining radius samples were fixed with 4% paraformaldehyde, and then embedded in methyl methacrylate and sliced to 40-μm-thick hard tissue sections. The calcein labeled fluorescence images were photographed by a CLSM and quantified by ImageJ software. The new bone formation rate was indicated by the mineral apposition rate (MAR, μm/day).

### Micro-CT scanning

The radius samples were analyzed by a Micro-CT Scanner (SkyScan 1076, Bruker, Belgium) at 90 kV voltage, 114 mA current, and 18 μm pixel size. The bone volume/total volume (BV/TV, %) of the repair regions can be quantitatively analyzed by a Micro-CT auxiliary software (NRecon version 1.6.6).

### Histological evaluation

Harvested bone samples were fixed in 4% paraformaldehyde and subsequently decalcified by Morse’s solution for 1 month. Subsequently, the samples were embedded for paraffin-sectioning to prepare sections about 5 μm. H&E staining was carried out according to the manufacturer’s instructions to observe new bone formation. Furthermore, the slices were blocked with 3% BSA in PBS containing 0.2% Triton X-100 for 1 h. Subsequently, the bone tissue sections were incubated with various primary antibodies, such as anti-Runx-2 (1:200), anti-Col-1 (1:200), anti-OCN (1:150), and anti-OPN (1:250) antibodies overnight at 4°C. The secondary antibodies, donkey anti-rabbit AlexaFluor 488 (1:300) and donkey anti-rat AlexaFluor 568 (1:300) were added and incubated with the slides for 1 h at room temperature. Finally, the sections were stained with DAPI and images were captured by CLSM.

### Statistical analysis

All results were expressed as mean ± standard deviation. All experiments were conducted independently at least 3 times. Comparisons among groups were analyzed with one-way ANOVA followed by Tukey’s post hoc test using SPSS 19.0 (SPSS Inc., Chicago, United States).

## Result and discussion

### Hydrogels preparation and characterization

**FIGURE 1:**
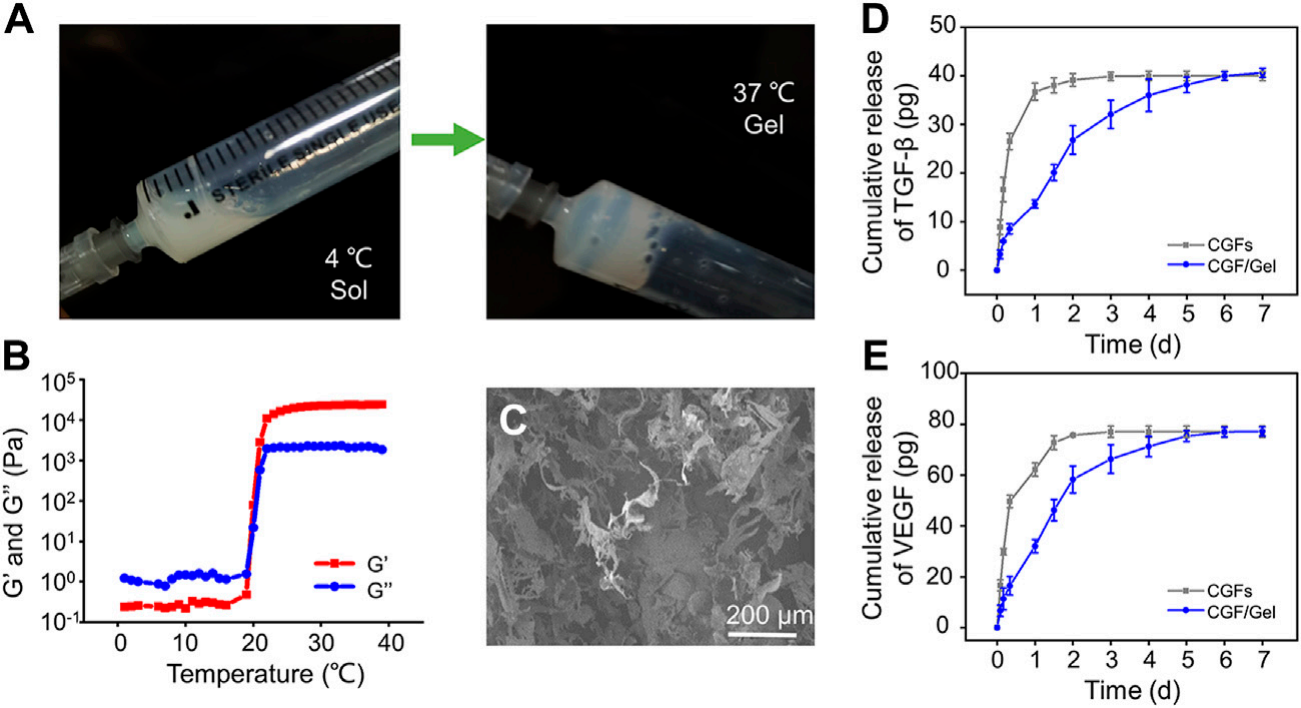
**(A)** The gross images of the sol-gel transition progress. **(B)** Rheometer detects the storage modulus (G′) and loss modulus (G″) of the hydrogel as the critical gelation temperature of the Poloxamer 407 hydrogel. **(C)** Morphologies of hydrogel observed by SEM. **(D)** The release profile of TGF-β in CGFs and and CGFs-loaded hydrogel (*n* = 3). **(E)** The release profile of VEGF in CGFs and and CGFs-loaded hydrogel (*n* = 3).

In recent years, diverse drug delivery systems have been developed for biomedical applications (Zhang et al., 2022a; Gao et al., 2022). Hydrogels have been widely used in biomedical fields because of their bionic three-dimensional (3D) network structure and loaded bioactive substances as drug delivery systems (Liu et al., 2018; Li et al., 2021b; Li et al., 2022c). Poloxamer 407 is a thermosensitive material with diversified capabilities, such as reversible thermo-sensitive property, drug encapsulation and sustained delivery ability, superior bioavailability, biocompatibility, and so on, which is considered to be an alternative injectable drug sustained-release system for enhancing bone regeneration and repair (Bai et al., 2020a; Chen et al., 2021). As illustrated in Figure 1A, the prepared hydrogel underwent a sol-gel transition with increasing the temperature from 4°C to 37°C. When the temperature dropped to 4°C again, the hydrogel can still be converted into a sol state. In low temperature, Poloxamer 407 presented as sol state, during which it can encapsulate bioactive substances for later sustained release from its gel state above critical gelation temperature (Beard et al., 2021). This reversible thermo-responsive performance of the Poloxamer 407 endows it to form hydrogel at body temperature and remain at the implantation site as a carrier for sustained drug release. To study the of value of critical gelation temperature of sol-gel transition, the rheological behaviors were investigated and demonstrated that both the storage modulus (*G′*) and loss modulus (*G″*) of the hydrogels were rapidly elevated after 22.1°C, suggesting that the specific critical gelation temperature of the Poloxamer 407 hydrogel was about 22.1°C (Figure 1B). Microstructure observation by SEM showed the hydrogel had interconnected pore structure (Figure 1C).

CGFs contain multiple growth factors, including TGF-β, VEGF, PDGF, FGF, IGF, and so on (Intini, 2009). Considering that the osteogenic induction of TGF-β and angiogenesis of VEGF play an important role in bone regeneration (Divband et al., 2021; Koyanagi et al., 2022), they were selected as representative factors of CGFs to study the release profiles of CGFs encapsulated in the hydrogel. As exhibited in Figures 1D,E, TGF-β and VEGF in the CGFs without hydrogel incorporating complete release within 24–36 h, showing a burst release rhythm. However, encapsulated the CGFs into Poloxamer 407 hydrogel, TGF-β and VEGF had stable release curves, which can last for 7 days. These results show that hydrogel can be used as a drug delivery system to deliver CGFs stably.

### Biocompatible hydrogels promote proliferation and maintain viability of BMSCs

**FIGURE 2:**
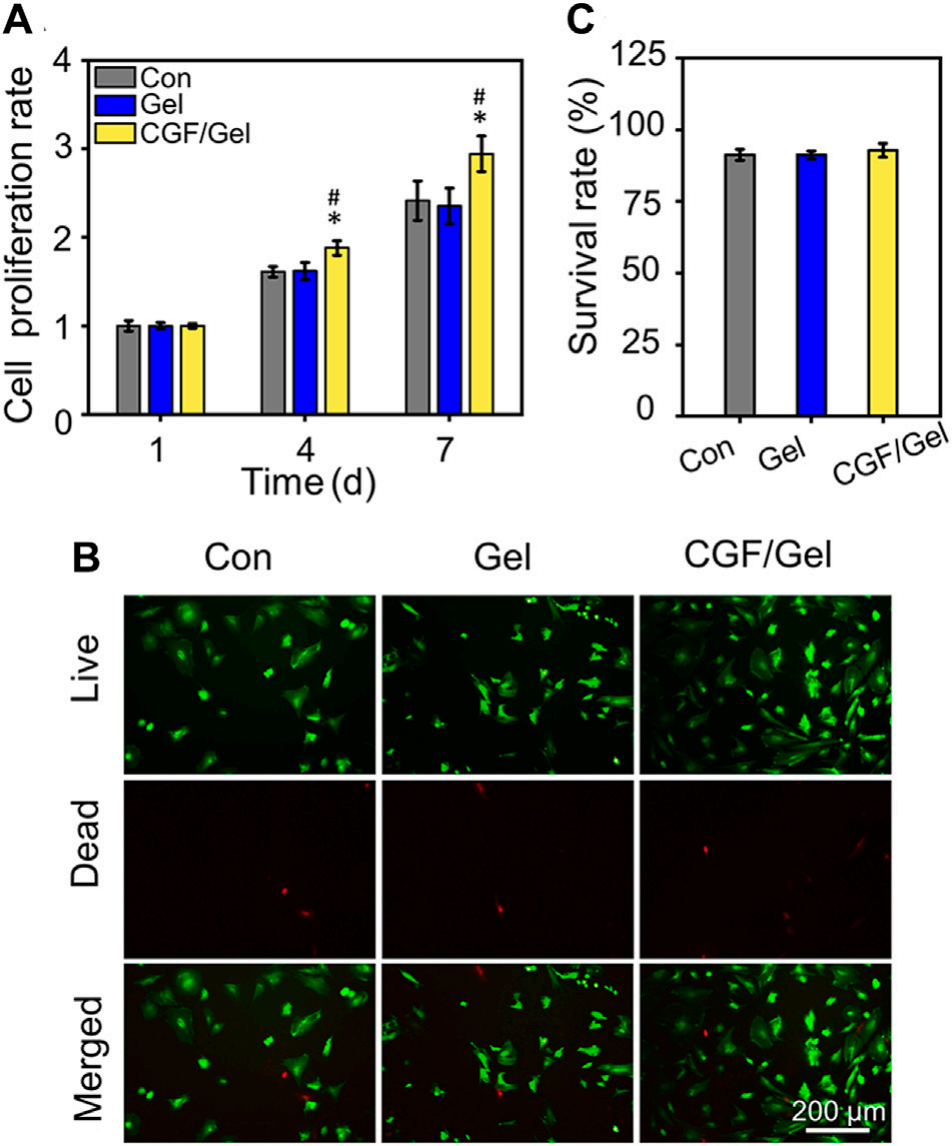
**(A)** Cell proliferation detection were evaluated by CCK-8 assay (*n* = 3). **(B)** Calcein AM/PI staining was conducted to mark the living and dead cells. **(C)** Quantitative analysis of the cell survival rates of the Con group, Gel group, and CGF/Gel group, which are 91.21% ± 1.95%, 91.22% ± 1.35%, and 92.77% ± 2.43%, respectively (*n* = 3) (*p < 0.05 compared with Con group; ^#^p < 0.05 compared with Gel group).

As shown in Figure 2A, with the prolonging of culture period, BMSCs in each group proliferated significantly, but the cell proliferation rate in CGF/Gel group was obviously higher than that in the Con and Gel groups on the 4th and 7th days (*p* < 0.05). Our results demonstrated that there was no significant difference among the Con group and the Gel group in showing that the Poloxamer 407 hydrogel had good biocompatibility. The superior proliferation rate of CGF/Gel group suggested that CGF-loaded hydrogel could promote cell proliferation. In addition, cell live and dead identification by Calcein AM/PI staining indicated that the cells were almost living cells stained with Calcein AM and maintained good survival rates in all groups (Figure 2B). Further quantitative analysis indicated that the survival rates of the Con group, Gel group, and CGF/Gel group were 91.21% ± 1.95%, 91.22% ± 1.35%, and 92.77% ± 2.43%, respectively (Figure 2C). These results suggested that the thermosensitive hydrogel had good biocompatibility and the CGFs-loaded hydrogel showed the capacity to promote BMSCs proliferation and maintain cell viability. BMSCs are a kind of adult stem cells derived from mesoderm, which have the potential of self-replication and multi-directional differentiation. They can differentiate into a variety of interstitial tissues, such as bone, cartilage, fat, bone marrow hematopoietic tissue and so on. The proliferation and differentiation of BMSCs are the basis of its function. Recently, BMSCs-based tissue engineering strategy is drawing widespread attention in bone regeneration and repair (Liu et al., 2013). When the bone injury occurs, BMSCs around the damaged site and circulation will migrate to the defect region and disperse into specific tissues. After a certain amount of stem cells are recruited to the damaged area, sufficient cell proliferation helps to provide more seed cells, thereby providing raw materials for subsequent directed differentiation and promoting the repair of tissue defects (Guo et al., 2020; Li et al., 2022e). Therefore, our CGFs-loaded hydrogel, which significantly promoted the proliferation of BMSCs, provide potential for subsequent critical-size bone defect repair.

### CGFs-loaded hydrogel promoted BMSCs osteogenic differentiation *in vitro*

**FIGURE 3:**
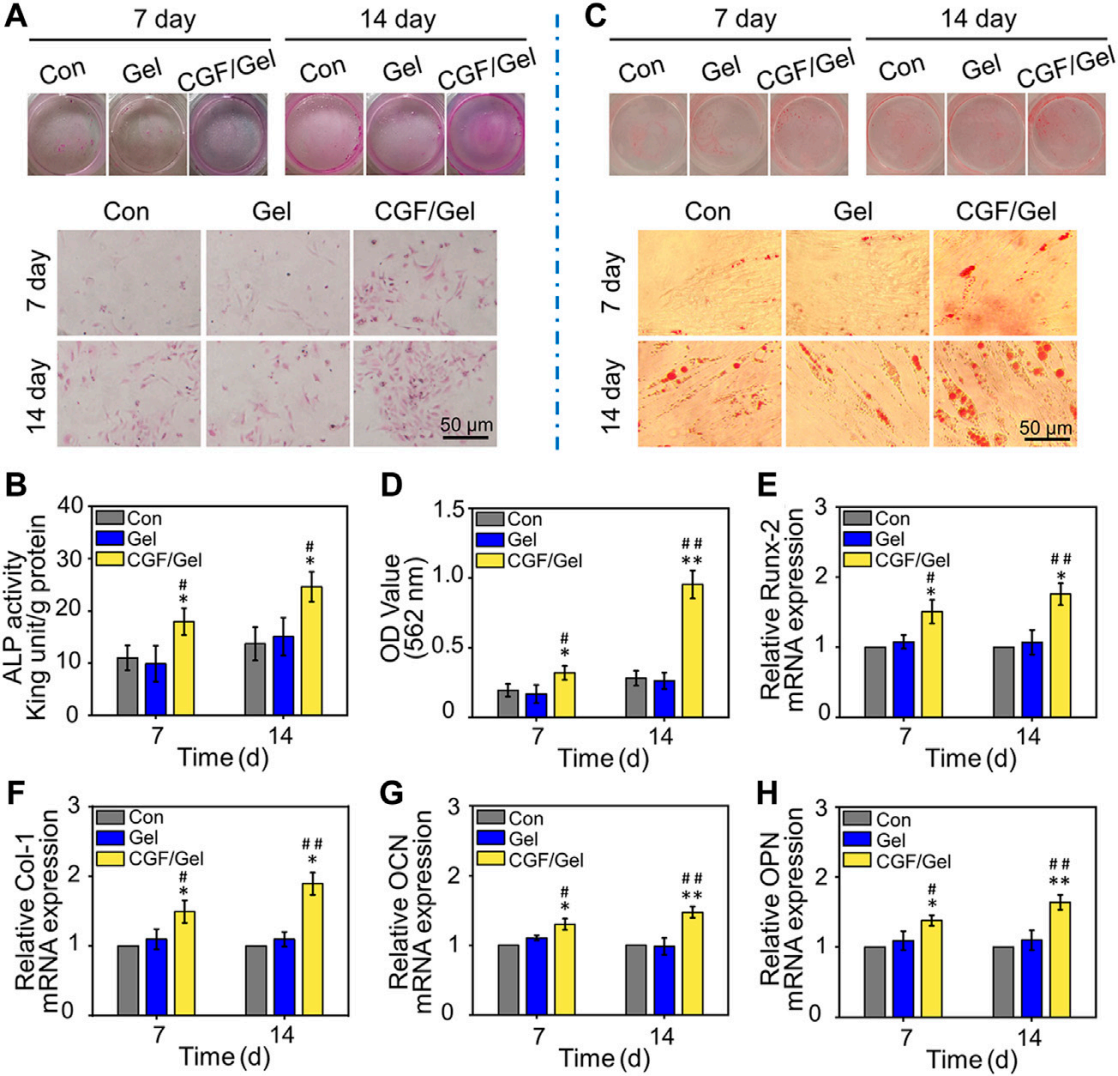
**(A)** Representative visual and microscopical images of ALP staining after 7 and 14 days of osteogenic induction. **(B)** Quantitative analysis of ALP activity of BMSCs (*n* = 3). **(C)** Representative visual and microscopical images of Alizarin Red staining to assess cell mineralization after 7 and 14 days of osteogenic induction. **(D)** Semi-quantitative analysis of alizarin red staining of BMSCs (*n* = 3). **(E–H)** The expression of osteogenic-related genes (*Runx-2*, *Col-1*, *OCN*, and *OPN*) in BMSCs after osteogenic induction for 7 and 14 days (*n* = 3) (*p < 0.05, **p < 0.01 compared with Con group; ^#^ *p* < 0.05, ^##^ *p* < 0.01 compared with Gel group).

The osteogenic differentiation activity of cells in the CGF-loaded hydrogel was evaluated by ALP activity detection, alizarin red staining, as well as RT-qPCR analysis with the expression of osteogenic differentiation genes. As shown in Figures 3A,B, after osteogenic induction culture for 7 and 14 days, the ALP activity investigation indicated that the ALP activity of the CGF/Gel group was obviously elevated than that of the Con group and Gel group at all time intervals (*p*＜0.05). Alizarin Red staining can mark the deposited calcium nodules to reveal the cell mineralization, which is a critical characteristic of osteogenic differentiation of BMSCs. As demonstrated in Figures 3C,D, gross images as well as semi-quantitative analysis showed that the CGFs-loaded hydrogel promoted the deposition of calcium nodules at the 7th day (*p*＜0.05) and the 14th day (*p*＜0.01).

In addition, to reveal the osteogenic induction effect of the CGFs-loaded hydrogel at gene level, some critical osteogenic markers (e.g., *Runx-2*, *Col-1*, *OCN*, and *OPN*) were detected by RT-qPCR assay. Runx-2 is considered one of the early osteogenic markers, which can induce subsequent osteogenic genes expression such as OCN and OPN (Bai et al., 2020b). As another important osteogenic differentiation marker, Col-1 is the premise of realizing the mineralization function of osteoblasts, as well as a major indicator suggesting the bone formation capacity (Zhao et al., 2020). In addition, OCN and OPN, which are considered important late osteogenic markers, are synthesized by osteoblasts and represent osteogenic maturation and bone formation (Qiao et al., 2020; Kim et al., 2021). In our study, the expression of these osteogenic marker genes in the CGF/Gel group were the highest, indicating that CGFs-loaded hydrogel provided with the best osteogenic induction for BMSCs (Figures 3E–H). These results demonstrated that the CGFs-loaded hydrogel played a significant role in promoting BMSCs osteogenic differentiation.

### CGFs-loaded hydrogel repaired critical-size bone defects efficiently

The critical-sized bone defects resulting from high-energy trauma, after extensive bone tumor resection, and severe bone infection, is a great challenge for orthopedics. Failure to repair these bone defects usually results in incomplete regeneration or nonunion, leading to disability. Developing various tissue engineered grafts have been considered as alternative strategies for bone healing and repair. To prepare an applicable tissue engineered graft demands a deep understanding of the primitive tissue to imitate the components and microstructure of native bone tissue, and the biological strategy of simulating the critical bioactive factors during bone repair process (Chu et al., 2021; Xie et al., 2021; Mou et al., 2022).

**FIGURE 4:**
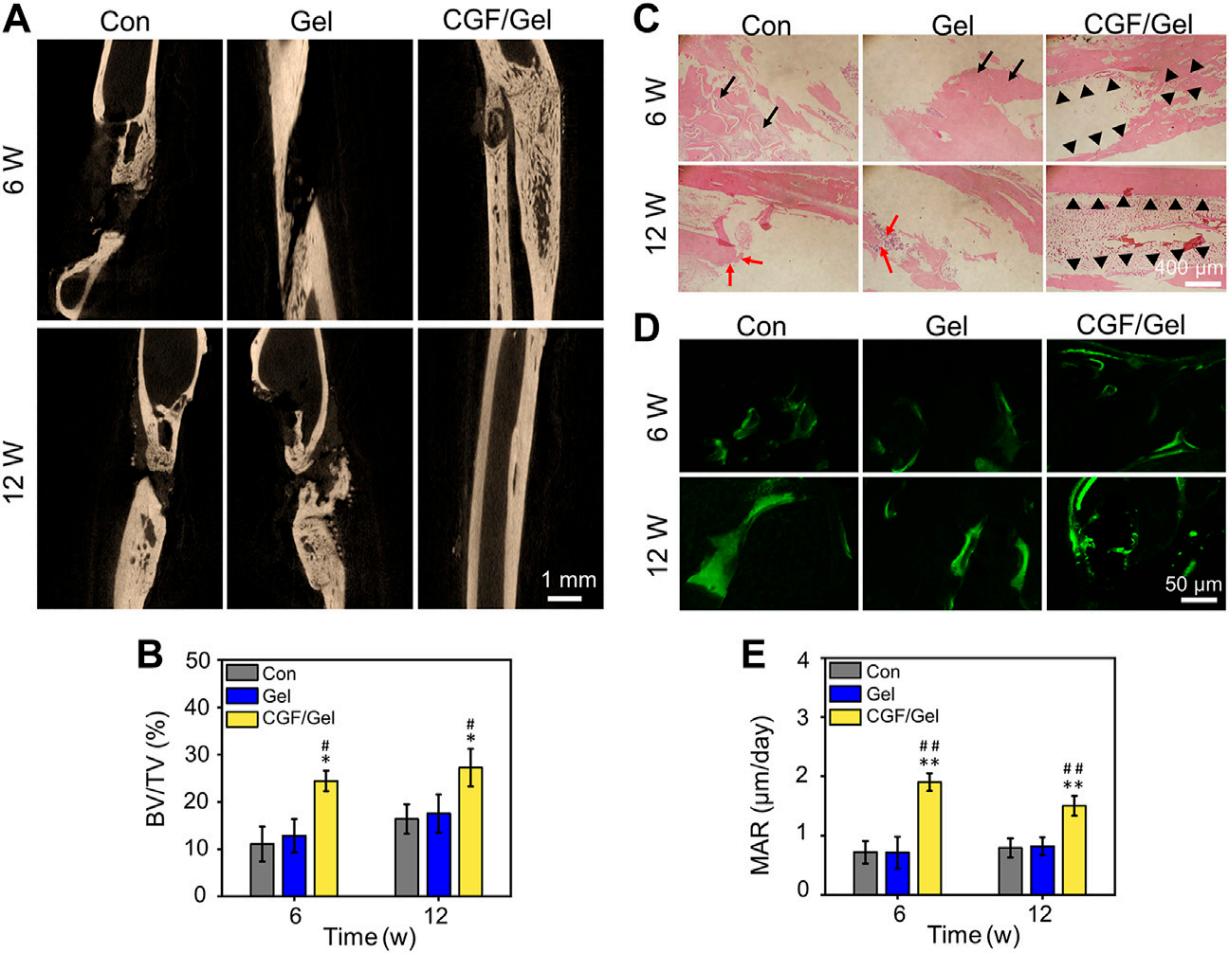
**(A)** Representative Micro-CT tomographic images of radius defects at the 6th and 12th week after surgery. **(B)** Quantitative analysis of bone morphometric parameter BV/TV (*n* = 3). **(C)** Histological H&E staining for observing bone regeneration in critical-size bone defects. Black arrows showed the fibrous tissue, red arrows indicated nonunion, and black triangles marked the regenerated bone tissue and continuous cortical bone structure. **(D)** Representative fluorescent images of calcein double-labeling in regenerated bone tissue. **(E)** Quantitative analysis of bone formation rate by MAR according to calcein double-labeling (*n* = 3) (*p < 0.05, **p < 0.01 compared with Con group; ^#^ *p* < 0.05, ^##^ *p* < 0.01 compared with Gel group).

CGFs is the third-generation platelet concentrate extracted and isolated from blood, which contains a large quantity and variety of growth factors, including PDGF, TGF-β1, TGF-β2, FGF, VEGF, IGF, and so on, and have been applied in clinical practice to promote tissue repair (Lokwani et al., 2020; Li et al., 2021a). CGFs, which does not contain bovine thrombin and anticoagulants, overcome the disadvantages in preparation and the high cost of platelet-rich plasma (PRP) and platelet-rich fibrin (PRF), as well as release various growth factors that promote cell proliferation and differentiation. Therefore, CGFs are regarded as ideal biological materials, which can partially address the limitations of traditional treatment strategies in critical-size bone defects regeneration (Li et al., 2021a). Previous studies have indicated that the combination of CGFs and bone substitute materials can significantly induce bone repair (Xu et al., 2019; Fang et al., 2020). To explore whether CGFs can promote the repair of critical-size bone defects effectively, we explored the healing efficiency of CGFs-loaded hydrogel *in vivo*. As exhibited in Figure 4A, the representative Micro-CT tomographic images displayed the defect zones with different treatments. Partial bone regeneration was observed in the Con group and Gel group, but the medullary cavity of the two fractured ends was closed, and finally bone nonunion occurred at 12th weeks after surgery. In order to further analyze the amount of bone regeneration in critical-size bone defects, the morphological parameters of regenerated bone were analyzed by Micro-CT software. Specifically, the BV/TV values of Con group, Gel group, and CGF/Gel group were 11.08% ± 3.72%, 12.86% ± 3.51%, and 24.43% ± 2.15% at the 6th week, while 16.39% ± 3.11%, 17.54% ± 4.05%, and 27.26% ± 3.96% at the 12th week, respectively (Figure 4B). Histological detection by H&E staining showed that the defect regions in Con group and Gel group were unhealed and filled with fibrous tissue, and nonunion was obviously observed at week 12. However, more bone tissues observed in the defects implanted with CGFs-loaded hydrogel, so as to obtain a continuous cortical bone structure (Figure 4C). In addition, as shown in Figure 4D, the fluorescent of calcein double-labeling was used to evaluated the MAR in the new formed bone. The evidence clearly demonstrated that the MAR value of CGF/Gel group was remarkable higher than those of the Con group and Gel group at 6 and 12 weeks after implantation (*p* < 0.01), indicating a faster new bone formation rate (Figure 4E). As evidenced by significantly enhanced bone morphological parameters, intact histological morphology and rapid mineral apposition, the CGFs-loaded hydrogel significantly promoted critical-size bone defects repair.

**FIGURE 5:**
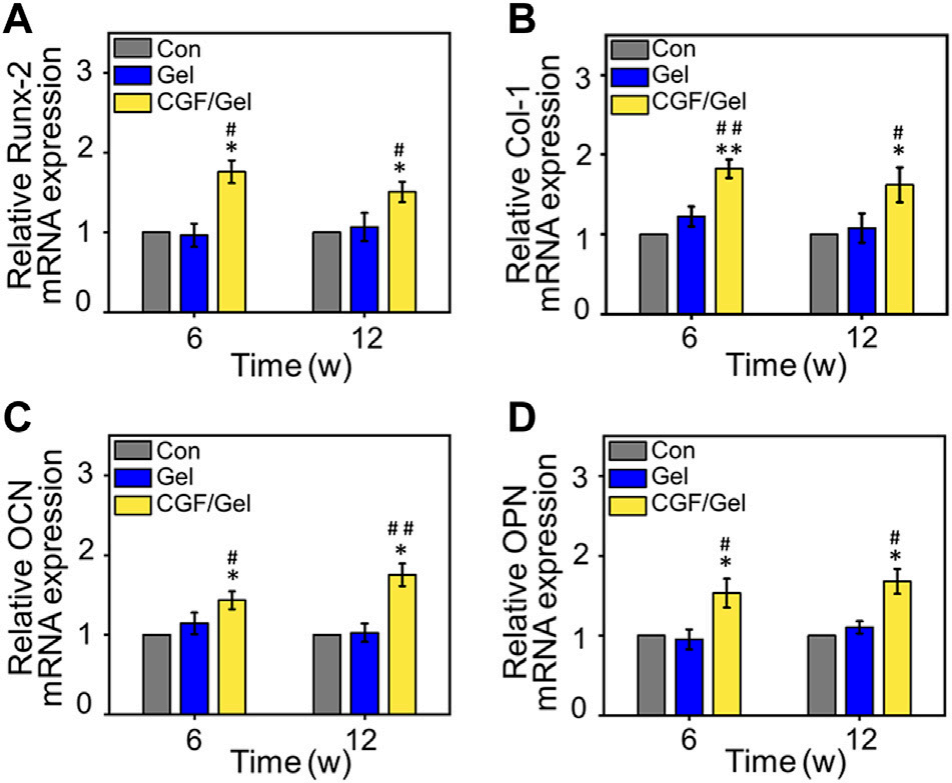
The mRNA expression levels of osteogenic-related genes, including **(A)** *Runx-2*, **(B)** *Col-1*, **(C)** *OCN*, and **(D)** *OPN* in the regenerated bone tissue (*n* = 3) (*p < 0.05 compared with Con group; ^#^ *p* < 0.05, ^##^ *p* < 0.01 compared with Gel group).

**FIGURE 6:**
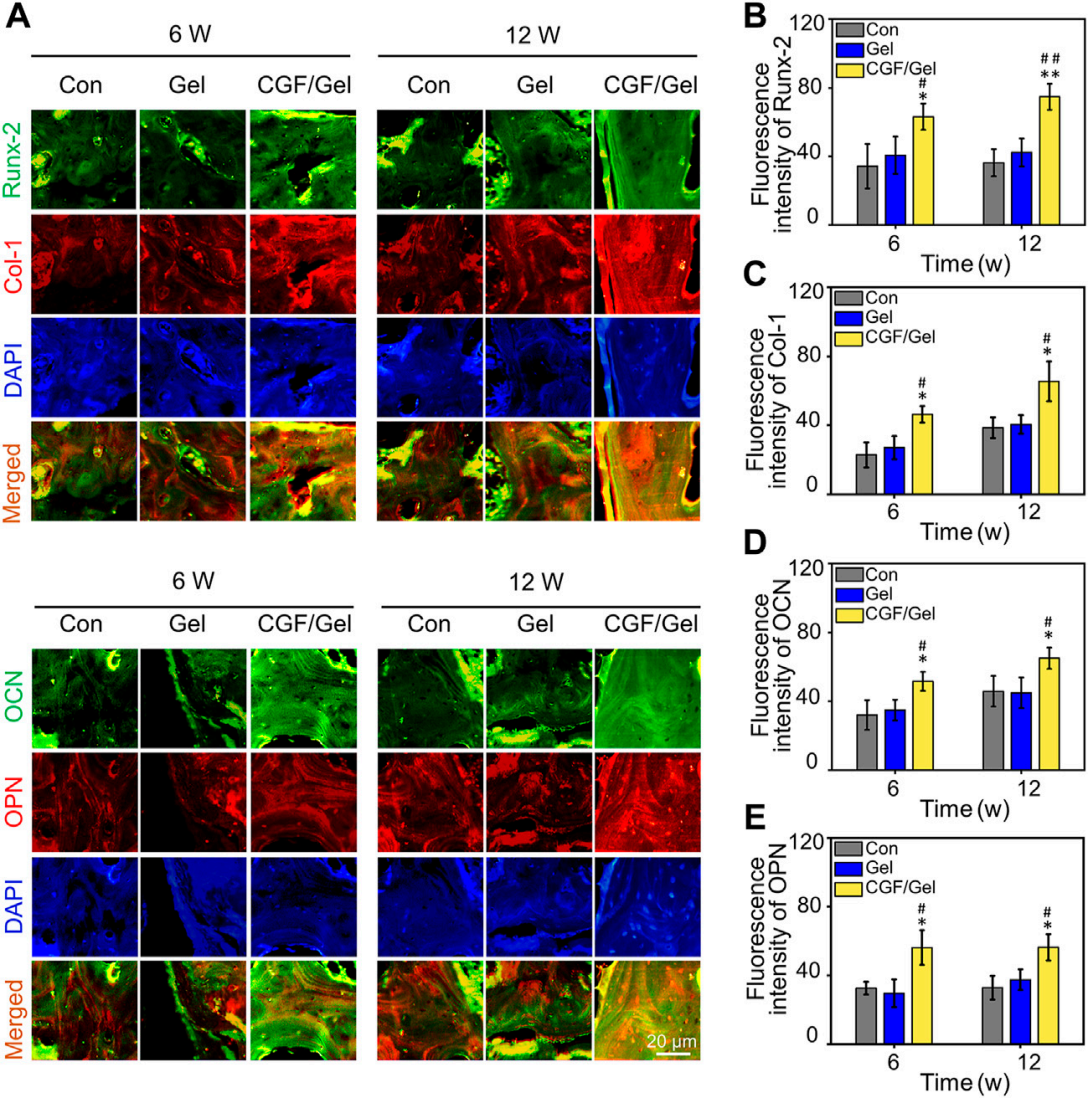
**(A)** Immunofluorescence staining of bone tissue in the critical-size defects. **(B–E)** Quantitative analysis of Runx-2, Col-1, OCN, and OPN according to the immunofluorescence (*n* = 3) (*p < 0.05, **p < 0.01 compared with Con group; ^#^ *p* < 0.05, ^##^ *p* < 0.01 compared with Gel group).

Subsequently, RT-qPCR analysis and immunofluorescence of osteogenesis-related markers were performed to study the mechanism of CGFs-loaded hydrogel promoting critical-size bone defects healing. RT-qPCR analysis indicated that osteogenic gene markers, including *Runx-2*, *Col-1*, *OCN*, as well as *OPN* in CGF/Gel group was strongly up-regulated compared with those of Con group and Gel group (Figures 5A–D). Furthermore, the expression of osteogenic differentiation associated proteins were observed with immunofluorescence methods (Figure 6A). Among these three groups, the fluorescence intensity of various proteins (e.g., Runx-2, Col-1, OCN, and OPN) in the CGF/Gel group showed the highest intensity in newly formed bone matrix (Figures 6B–E).

Autologous blood derived products, such as CGFs and PRP, are concentrated mix of multiple growth factors and cytokines. These products have been well documented for their potential capacity to promote tissue repair. But with direct use of these blood concentrating substances locally at the injury sites, the burst release of encapsulated active factors can lead to excessive local concentrations with a non-sustained duration of action (Jain et al., 2017). Therefore, it is expected to obtain a better tissue repair effect by incorporating these blood concentrates rich in growth factors into the drug release system to obtain a continuous release profile (Zhang et al., 2022b). For example, Kakudo *et al.* introduced PRP into the biodegradable gelatin hydrogel to provide controlled-release effects of various growth factors, thus achieving better vascular regeneration and tissue repair effects than using PRP alone (Kakudo et al., 2017). In this study, CGFs containing thermosensitive hydrogel could show sustained growth factor release profiles, promote osteogenic differentiation of BMSCs to a greater extent, as well as improve critical-size bone defects repair *in vivo* by up-regulating osteogenic-related markers, such as Runx-2, Col-1, OCN, and OPN. The preparation process of this composite of CGFs with Poloxamer 407 hydrogel is simple and easy to batch produce, thus providing an effective therapy for repair critical-size bone defects.

## Conclusion

We designed a CGFs-loaded hydrogel with temperature sensitive performance and biocompatibility. This hydrogel provided a satisfactory result of promoting BMSCs proliferation and maintain viability, as well as inducing osteogenic differentiation. In summary, the CGFs-loaded hydrogel significantly induced critical-size bone defects healing and prevent the occurs of nonunion. This therapy strategy provides a novel efficacious strategy for enhancing critical-size bone defects repair.

## Data Availability

The data that support the findings of this study are accessible from the corresponding author upon reasonable request.
